# Hay Fever

**Published:** 1872-02

**Authors:** 


### HAY FEVER.

This is a troublesome ailment, with which some persons are afflicted every year, coming on with great regularity, sometimes the same week and the same day. It is a kind of a summer cold, with sneezings, watery eyes, running from the nose, and sometimes the breathing becomes affected, with tightness across the forehead, or in the chest. Medicine seems to have no good effect whatever. Whether it arises from the dust of hay, or flowers, or plants, is not positively known. At the same time, persons have escaped it by going to the sea-shore, or to mountain localities; perhaps a visit to the North Pole during August might prevent an attack, and if repeated several years in succession, might break up the habit of it, and thus effect a permanent cure. A distinguished clergyman escaped an attack last summer, by spending it among the White Mountains, at the Twin Mountain House, a few miles from the "Tip-top" House; and a very pleasant hostelry it is, as we know by experience.

